# The interplay between different potassium electrolytes and MoS_2_@SiC@S cathodes in the performance of potassium–sulfur batteries

**DOI:** 10.1039/d4ra06101b

**Published:** 2024-11-27

**Authors:** Li Zhou, Osama Ragab, N. K. Wally, Khaled Faisal Qasim, Xuejin Li, M. M. El-Desoky, Wei Xing, E. Sheha

**Affiliations:** a School of Materials Science and Engineering, Advanced Chemical Engineering and Energy Materials Research Center, China University of Petroleum (East China) Qingdao 266580 China; b Physics Department, Faculty of Science, Benha University Benha 13518 Egypt islam.shihah@fsc.bu.edu.eg; c Physics Department, Faculty of Science, Suez University Suez 43518 Egypt; d Chemistry Department, Faculty of Science, Suez University Suez 43518 Egypt

## Abstract

Potassium–sulfur batteries (KSBs) have garnered immense attention as a high-energy, cost-effective energy storage system. Nevertheless, achieving optimal sulfur utilization and long-term cycling is the primary challenge. To address some of the different challenges of KSBs, a potassium–sulfur (K/S) battery cathode was fabricated using MoS_2_, SiC, and S (Mo@Si@S) in a fixed ratio. The powder was characterized using X-ray diffraction (XRD), scanning electron microscope (SEM), thermogravimetric analysis (TGA), and energy dispersive X-ray (EDX) mapping analysis. Coin cells were fabricated using K metal as an anode, and three different electrolytes were used to determine the effect of electrolytes on electrochemical performance. The battery using the KPF_6_ electrolyte displayed the best electrochemical performance with a 713 mA h g^−1^ capacity compared to the other two electrolytes. The structure and morphological evolution of pristine, charged, and discharged states were explored using XRD, SEM, and EDX mapping. The results showed that sulfur was successfully diffused within the cathode, and K was homogeneously distributed, suggesting the good performance of the modified electrode.

## Abstract 2

Potassium–sulfur batteries (KSBs) have garnered immense attention as a high-energy, cost-effective energy storage system.

## Graphical abstract

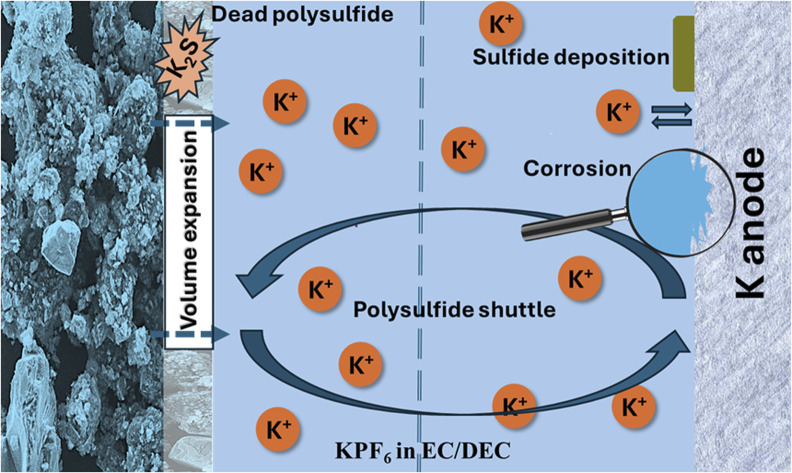

## 1. Introduction

The potassium–sulfur (K/S) battery represents an innovative energy storage technology, leveraging potassium (K) and sulfur (S) as core components in its chemical composition. Similar to lithium–sulfur (Li–S) batteries, the K/S battery substitutes potassium ions (K^+^) for lithium ions (Li^+^).^1^ This technology is being actively explored due to the more widespread availability and lower cost of potassium than those of lithium, potentially offering a more sustainable and economically viable energy storage solution.^2^ Belonging to the broader category of metal–sulfur batteries, K/S batteries are attracting attention for their high energy density and the abundant resources used in their production.^3^ The K/S cathode operates on the potassium–sulfur (K–S) chemistry principle, involving multi-electron transfer reactions that promise high-capacity energy storage. Optimization efforts in battery design primarily focus on enhancing the performance of the cathode, anode, and separator to mitigate challenges such as the polysulfide shuttle effect. Improving the cycle life of K/S batteries presents a significant challenge due to the gradual degradation of cathode materials through repeated charge and discharge cycles. Safety is also critical, given potassium's high reactivity, necessitating careful management to mitigate potential risks.^4^

In K/S batteries, MoS_2_ plays a crucial role as the cathode material due to several key characteristics. Its high electrical conductivity facilitates efficient electron transfer during the battery charge and discharge cycles, optimizing overall performance and efficiency. MoS_2_ exhibits chemical stability in the presence of potassium polysulfides, which are intermediate compounds formed during electrochemical reactions in K/S batteries. This stability helps prevent the dissolution of active materials and preserves battery performance over numerous cycles.^5^ Additionally, MoS_2_ acts as a catalyst in converting polysulfides generated during discharge into simpler sulfur species. This catalytic activity effectively counters the “shuttle effect”, a common issue in sulfur-based batteries where polysulfides dissolve in the electrolyte and migrate, causing capacity loss. Moreover, Mo compounds are abundant and relatively cost-effective compared to other metals used in battery cathodes, contributing to the potential cost-effectiveness of K/S batteries.^6^ Also, Mo is environmentally friendly, being a necessary element for both animals and plants, which makes Mo a promising element for several applications, such as a recent catalyst for the oxidative deoximation reaction.^7^ Prior studies have demonstrated the significance of adding catalytically active sites within sulfur hosts to promote the K/S conversion. Recently, Saroja *et al.*^8^ employed commercial MoS_2_ in a K/S battery in 1 M bis(fluorosulfonyl)imide potassium salt (KFSI) in 1 : 1 of ethylene carbonate (EC) and diethyl carbonate (DEC). They used in-depth characterization techniques to study the charge storage mechanism, where they detected dual redox activity. They indicated that an irreversible Mo oxidation induces the reaction pathway to be redirected towards S oxidation, which promotes K/S electrochemistry during the (de)potassiation step. However, nanoscale MoS_2_ significantly agglomerates and exhibits low conductivity, which results in low charge transfer and reduced performance. Fortunately, the ability of nanomaterials to convert energy can be improved by altering their characteristics through element doping. Modulating the MoS_2_ lattice by incorporating atoms with different electronegativities can expose more active sites and improve its chemical activity.^9^

Song *et al.*^2^ have synthesized a W_SA_–W_2_C@NC/S cathode material with combined functionalities. Tungsten single atom (W_SA_)-modified carbon substrate is a fast route for rapid potassium polysulfide migration. This approach was used to address the issue of mismatching in sulfur and catalytic site distribution. On the other hand, W_2_C nanocrystals offer separate catalytic sites for potassium polysulfide conversion. The polysulfides could be supplied to W_2_C for catalytic conversion during the cycling processes, and the reaction products can then quickly migrate from W_2_C sites.

Silicon carbide (SiC) as a cathode material in potassium–sulfur batteries offers several significant advantages that enhance overall performance and stability. Its chemical stability and corrosion resistance, particularly in the presence of potassium polysulfides formed during charge and discharge cycles, prevent the dissolution of active materials and reduce side reactions that could degrade battery performance over time.^10^ SiC is renowned for its excellent electrical and thermal conductivity, which is crucial for efficient electron transfer during electrochemical processes in the battery. This property supports high efficiency, minimizes internal resistance, and thereby improves overall battery performance.^11^ The high thermal conductivity of SiC facilitates effective heat dissipation during battery operation, which is crucial for preventing overheating and thermal runaway. This feature enhances safety and prolongs the lifespan of K/S batteries.^12^ Moreover, SiC boasts a high theoretical capacity, allowing it to store substantial energy per unit mass. This characteristic is advantageous for boosting the energy density of potassium–sulfur batteries, making them suitable for applications requiring high energy storage capacities.^13,14^

The development of K/S batteries aims to address challenges, such as high energy density, long cycle life, and environmental impact, making them a significant area of interest in advancing battery technology for future applications. To our knowledge, there is no previous research where SiC was employed as an electrode material for potassium–sulfur batteries, although it has inherent success in the electrodes of lithium-ion batteries.^15–18^ A simple microwave preparation strategy was employed to obtain a particular cathode nanomaterial containing S, MoS_2_, SiC, and C as a cathode candidate for future potassium-ion batteries. Then, Mo@Si@S is characterized by differential scanning calorimeter (DSC), thermogravimetric analysis (TGA), X-ray diffraction (XRD), scanning electron micrograph (SEM), and energy dispersive X-ray spectrometry (EDS) for sample structural and morphological characterization and analysis. Finally, the SiC–MoS_2_ cathode is used as an active material in 2032-type cells with three different electrolytes, and galvanostatic and cyclic-voltammetry tests record electrochemical performance to evaluate Mo@Si@S cathode performance in the K/S battery and electrolyte compatibility.

## 2. Experimental technique

### 2.1 Cathode preparation and characterization

The Mo@Si@S cathode was prepared as follows: 30 wt% Super P carbon (MTI A World Leader in Advanced Material & Laboratory Equipment), 30 wt% sulfur powder (S, Alfa Aesar 99%), 20 wt% silicon carbide (ALDRICH 200 mesh particle size) and 20 wt% molybdenum(iv) sulfide (ALDRICH 99%) were mixed well in an agate mortar to ensure uniform dispersion. Then, the reactant mixture was microwaved for 10 seconds with a pulse of 1200 watts. To prepare the cell electrode, 0.75 Mo@Si@S : 0.15 g poly(vinylidene fluoride) powder (–CH_2_CF_2_–)_*n*_ were dissolved in *N*-methyl-2-pyrrolidinone solution to create a thick fluid using a magnetic stirrer. The resulting thick fluid was distributed using a HOHSEN MC-20 Mini-Coater over 150 mm of Al foil, and the film of the cathode was left in the oven at 100 °C for two hours to dry well. Then, the cathode was cut with a diameter of 14 mm. It was maintained at 55 °C in the incubator to keep moisture from getting to the cathode. The crystal structures were characterized by X-ray diffraction (XRD, Empyrean) with Cu Kα radiation. Linseis TGA PT1000 was used for differential scanning calorimetry (DSC) and thermogravimetric analysis (TGA). The morphologies and energy dispersive spectrometry (EDS) analysis were obtained by scanning electron microscopy (SEM, HITACHI Regulus8100).

### 2.2 Cell assembling and electrochemical tests

Self-made electrolyte-based preheated aluminum nitrate salt at 80 °C was used to remove the moisture from molecules dissolved in 5.4 : 2.7 mL of acetonitrile (ACN) and tetraethylene glycol dimethyl. The electrochemical behavior and performance were examined by assembling 2032-type cells. A thin sheet of potassium pressed from a potassium block on a stainless-steel spacer was used as the anode. The separator was the glass fiber filter membrane (1827-0470, Whatman). The electrolyte includes three types: 1 M KFSI/EC–DEC, 0.8 M KPF_6_/EC–DEC, and the self-made electrolyte. The 2032-type cells were assembled in a glove box filled with an argon atmosphere (H_2_O and O_2_ content constant below 0.1 ppm) and subjected to electrochemical testing within 0–2.2 V (*vs.* K^+^/K). Cyclic voltammetry (CV) curves at various scan rates were performed by the Bio-Logic electrochemical workstation. The specific capacity and cycling stability were measured using the LANBT test instrument.

## 3. Results and discussion

### 3.1 X-ray diffraction (XRD)

**Fig. 1:**
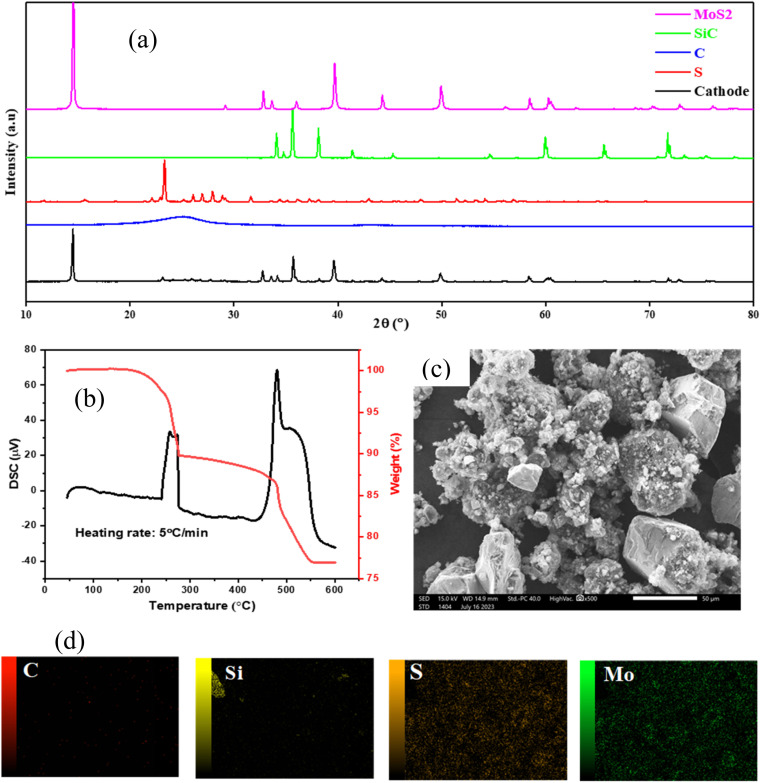
(a) XRD, (b) TGA & DSC, (c) SEM, (d) EDX mapping for the used powder as a cathode.

The XRD patterns of raw materials are shown in Fig. 1a, where the MoS_2_ pattern is matched with hexagonal (JCPDS #006-0097) with *P*6_3_/*mmc* space group. SiC is matched with rhombohedral (JCPDS #002-1048) with *R*3*m* space group, while the S pattern can be well matched as orthorhombic (JCPDS #008-0247) with space group *Fddd*. The XRD pattern of the prepared Mo@Si@S cathode material is also displayed in Fig. 1a with no peak position or relative intensity changes, indicating no phase transformations occurred. However, some peaks were suppressed due to the presence of higher-intensity hexagonal MoS_2_ peaks. The average crystallite size of the cathode material is calculated according to the Debye–Scherrer equation;^19^1
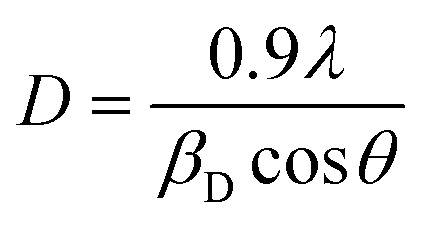
where *λ* denotes the wavelength of Cu Kα (given as 1.542 Å), and *β*_D_ denotes the corrected full width (calculated in radians values) at half maximum. The average crystallite size is found to be 49 nm. These findings show that the intended cathode nanomaterial was successfully prepared.

### 3.2 Differential scanning calorimetry (DSC) and thermogravimetric analysis (TGA)

DSC and TGA of Mo@Si@S cathode are studied to analyze the thermal stability of the as-prepared SiC–MoS_2_ cathode. Fig. 1b displays the recorded DSC and TGA data. The DSC pattern exhibits three significant exothermic peaks at 260, 275, and 480 °C. These peaks coincided with similar changes in the mass of the SiC–MoS_2_ cathode, as specific weight losses in the TGA thermogram were evident at the same temperatures. The initial drop can be due to the extraction of water molecules from the cathode surface.^20^ This weight loss and the corresponding DSC peak are evidence of sulfur evaporation starting at 260 °C and positioned at 275 °C. The S content determined by TGA (10%) is not the same as the starting raw materials, which can be due to sulfur loss during the preparation step, as previously reported.^21^ The final weight drop is found in the 475–550 °C range and is ascribed to carbonaceous material decomposition with a peak temperature located at 480 °C. This temperature exceeds the previously reported value,^22^ which can be ascribed to the MoS_2_, SiC, and S incorporation. There was no evidence of MoS_2_ to MoO_3_ conversion at 400 °C.^23^ The remaining 77% of the cathode material mass can be deduced as MoS_2_, SiC, and carbonaceous degradation products. Moreover, no noticeable weight loss was evident beyond 550 °C, and the weight loss up to 600 °C was only 23%. These findings suggest that the current cathode is thermally stable at temperatures reaching 260 °C. As a result, the synthesized SiC–MoS_2_ cathode can be employed as an electrode in rechargeable batteries without experiencing a significant reduction in performance, even at such high temperatures.

### 3.3 Scanning electron microscopy (SEM) and energy dispersive spectrometry (EDS)

Fig. 1c shows the SEM image of the morphology of the Mo@Si@S cathode material. MoS_2_ can be seen as the well-known irregular flower-shaped spheres.^24,25^ The size of these particles is in the range of 50–55 μm. They are homogeneously covered with a Super P conductive carbon layer that appears granular with a slightly rough surface texture.^26^ This is in favor of the large surface area available for electrochemical reactions to occur and facilitates the electron and ion transport within the cathode active material, which is vital for battery performance.^27^ SiC appeared as large irregular particles having a size in the range of 65–75 μm.^28^ Small sulfur particles were scattered across the sample with high homogeneity. This well-dispersed composite structure is essential to sustain a uniform electrode morphology while reversible-phase transformation occurs during the potassium–sulfur battery's cycling processes. Fig. 1d shows the elemental mapping of C, Si, S, and Mo, which is compatible with SEM findings where all elements are uniformly distributed across the samples, as seen in Table 1.

**Table 1:** Elemental ratio of the prepared materials

Sample	SEM-EDX (atomic%)				
	Mo	S	Si	C	K
Powder	15.15	27.7	1.75	55.35	—
Pristine	12.18	9.18	5.05	73.53	0.03
Charged	18.59	5.3	4.72	45.7	25.7
Discharged	10.09	4.57	3.53	53.36	28.45

### 3.4 Electrochemical measurements

**Fig. 2:**
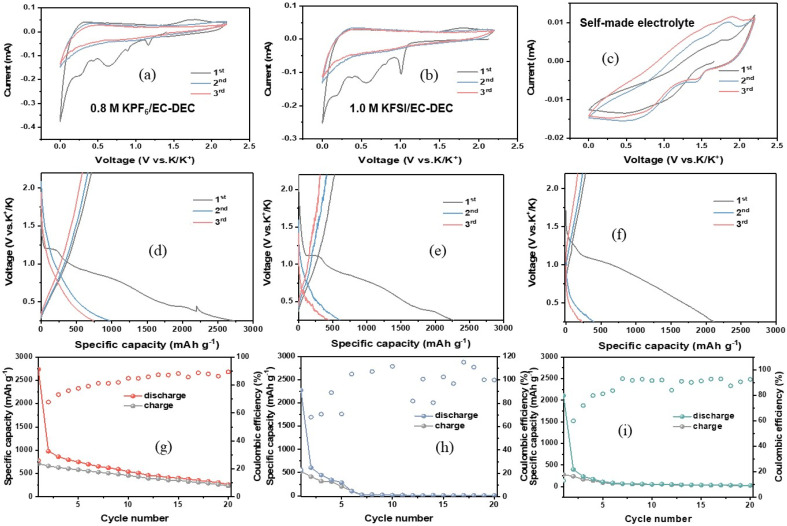
The capacity results of (a–c) CV, (d–f) GCD, and (g–i) cycle stability for 3 electrolytes.

The electrochemical performance of the Mo@Si@S containing composites was evaluated using different techniques. Fig. 2 presents the cycle performance and cyclic voltammetry of Mo@Si@S through 3 electrolytes. CV of Mo@Si@S at a scan rate of 100 μV s^−1^ in the range of 0–2.5 V in Fig. 2a–c shows different hysteresis with changing the electrolyte. The capacity and stability of Mo@Si@S vary with electrolyte changes because of the variation in the K content, according to the following order: KPF_6_ > KFSI > self-made electrolyte. Interestingly, the KPF_6_ electrolyte delivers the highest capacity, possibly due to the suitable sulfur content and high conductivity. The KPF_6_ electrolyte peaks at 0.26, 0.44, 0.66, 0.9, and 1.16 V are related to K_2_S_2_, K_2_S_3_, K_2_S_4_, K_2_S_5_, and K_2_S_6_.^29^ The KFSI electrolyte peaks at 0.18, 0.38, 0.56, 0.81, and 1 V are related to K_2_S_2_, K_2_S_3_, K_2_S_4_, K_2_S_5_, and K_2_S_6_, respectively. Although batteries using KPF_6_ and KFSI show relatively high capacity (2994.2 mA h g^−1^, 2399.3 mA h g^−1^, respectively), they have a demolition of redox peaks after the 1^st^ cycle, owing to the polysulfide diffusion within the Mo@Si@S electrode.^30^ The battery with the self-made electrolyte shows the redox peaks at 0.65, 1.42, and 1.84 V, which are related to K_2_S_2_, K_2_S_4_, and K_2_S_6_, respectively. The self-made electrolyte shows the stability of redox peaks even after the 3^rd^ cycle of the Mo@Si@S electrode (from 198.2 to 343.6 mA h g^−1^). However, as seen from Fig. 2c, the redox voltage of the self-made electrolyte is shifted towards higher voltage in the 3^rd^ cycle, which is related to bad cycle stability owing to the growth of the interfacial barrier and polysulfide shuttle.^31,32^ Hence, stability decreased, as seen in Table 2. Fig. 2d–f shows galvanostatic curves of K/S cells with varying electrolyte systems; all cells delivered high irreversible initial discharge capacity > 2000 mA h g^−1^, which confirms partial electrolyte decomposition in forming a solid electrolyte interface. Subsequently, K/S cells show stable charge/discharge curves with a capacity > 2000 mA h g^−1^. Using the KPF_6_ electrolyte, it shows that humps appear at 1.2, 0.8, and 0.55 V, while the KFSI electrolyte's humps are at 1.1, 0.75, and 0.4 V. The self-made electrolyte has humps at 1.25 and 1 V. All those humps are related to the polysulfide intercalation in the Mo@Si@S electrode. The battery using the KPF_6_ electrolyte displays the best electrochemical performance with a 713 mA h g^−1^ capacity compared to the other two electrolytes, as shown in Fig. 2g–i. The difference in capacity when changing the electrolyte is related to the higher ionic conductivity of KPF_6_ (∼10–15 mS cm^−1^) *versus* KTFSI (5–10 mS cm^−1^), which can lead to more efficient charge transport and higher capacitance in electrochemical systems. The higher ionic conductivity is related to several factors like ionic size, which is lower in PF_6_^−^, which increases ionic mobility. Also, the relatively lower viscosity and density of KPF_6_ affect the interfacial properties that enhance both capacitive and diffusive capacity. Besides, the electrochemical stability of KPF_6_ at higher voltages (∼5 V) rather than KTFSI (∼4–4.5 V), thus increases the electrochemical stability.

**Table 2:** The capacity of the used electrode for different electrolytes

Electrolyte	Capacity from CV at 100 μV s^−1^ (mA h g^−1^)			Capacity from GCD at 0.3 A g^−1^ (mA h g^−1^)					
	1^st^ cycle	2^nd^ cycle	3^rd^ cycle	1^st^ cycle		2^nd^ cycle		3^rd^ cycle	
				Charge	Discharge	Charge	Discharge	Charge	Discharge
KPF_6_	2994.2	1533.2	1424.96	713.1	2735	662.3	977.7	581	750.1
KFSI	2398.9	1316.7	1172.4	525	2270	412	605	313	445
Self-made	198.4	288.6	342.7	277.8	2109	232.6	395.6	167.5	237

**Fig. 3:**
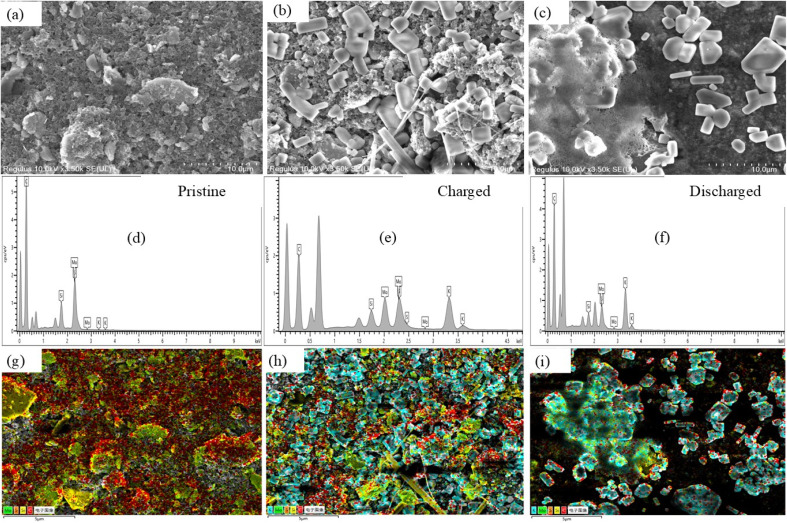
(a–c) SEM, (d–f) EDS, and (g–i) mapping of the Si@Mo@S sample in pristine, charged, and discharged forms.

**Fig. 4:**
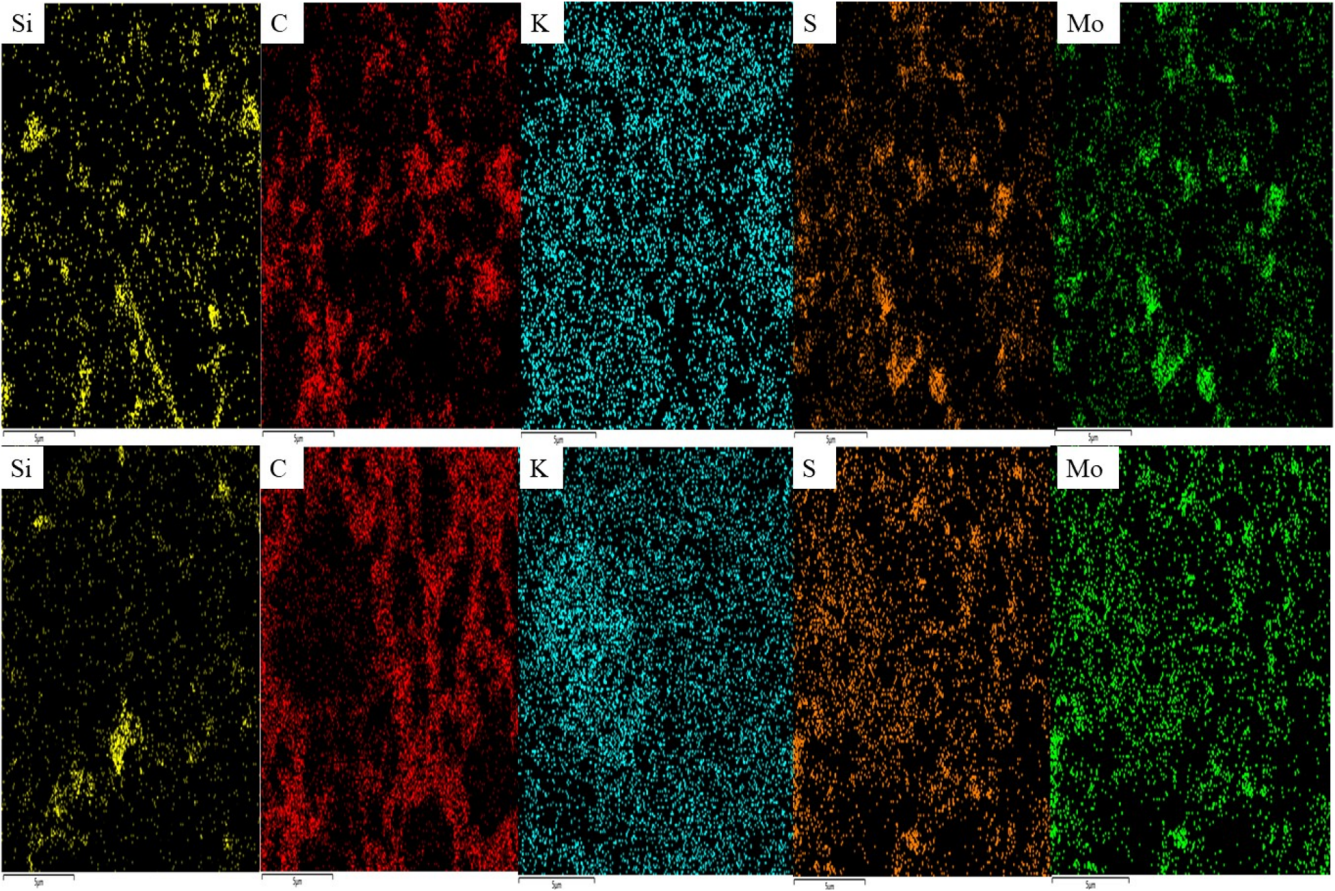
EDS mapping of the charged (top) and discharged (down) Si@Mo@S electrode.

To evaluate the effect of the charge/discharge process on the evolution of the morphological and structural aspects of Mo@Si@S, K/S coin cells were disassembled, and the extracted Mo@Si@S discs were washed and dried for further study on the surface side that was in contact with the electrolyte. X-ray diffraction (XRD), elemental mapping, and high-resolution SEM (HRSEM) were conducted for a more intuitive understanding of the electrochemical mechanism during the potassiation/de-potassiation (K^+^/de-K^+^) process. SEM images, EDS, and mapping of pristine, charged, and discharged discs are illustrated in Fig. 3. Fig. 3a, d and g present the pristine state of the Mo@Si@S electrode; it is found that the distribution of C, S, and Mo is highly uniform and well-distributed on the surface, while the majority of Si is in bulk. All SEM images in Fig. 3a–c show the same morphology with different particle sizes owing to agglomeration during the electrochemical process, indicating the successful de-K^+^ during the charging process. The EDS spectra of the three electrodes are shown in Fig. 3d–f; after the charging process started, the K content increased significantly, although the charging process corresponded to the de-K^+^ process, which decreased the potassium content. This unexpectedly higher potassium content at the charged state in Fig. 3e may be due to the electrolyte remaining on the electrode or a partially irreversible reaction. Additionally, the dramatic decrease in the S ratio is related to the electrochemical reaction occurring on S by forming potassium polysulfide (K_2_S_*x*_), followed by diffusion within the electrode,^32^ as shown in Fig. 3e and f. The atomic ratio of S : K is considered to be 1 : 4.84 during the charging process. However, during the discharging process, the homogeneity of S, C, and Mo returned, keeping the low homogeneity of Si and the same homogeneity of K. As seen in Table 1, there is an observed increment of K%, with the Mo and S% decrement during discharge, and the ratio of S : K reaches 1 : 6.22 (higher than charge). It shows that K ions shuttle to the cathode and diffuse in Mo@Si@S, followed by polysulfides (K_2_S_*x*_) made sulfur shut; hence, the MoS_2_ structure has been demolished.^4,33–35^ To study the mechanism of SEI evolution in KFSI and KPF_6_ dissolved in EC/DEC electrolytes, Deng *et al.*^36^ assembled half-cells of K‖MoS_2_. After careful examination, they confirmed that a SEI layer was formed on MoS_2_ in both electrolytes, albeit with different compositions. Using a self-made electrolyte, the SEI was formed with a KF-rich layer. On the other hand, a KF-deficient layer is formed in a KPF_6_-based electrolyte where the layer is rich in organic species due to the higher decomposition stability of PF_6_^−^ in KPF_6_ compared to FSI^−^ in KFSI. As a result, the formed SEI layer in the KPF_6_-based electrolyte was mainly composed of decomposition products of organic solvents. These products do not have the ability to passivate MoS_2_ and hinder its reactivity with electrolytes; hence, electrolyte decomposition and electrode capacity decay occur. In the current work with KPF_6_ electrolyte, the elemental ratio of Mo@Si@S surface shows the increase in K ratio after cycling. However, no increase in the C ratio is detected, which suggests that no decomposition products of organic solvents are present on the electrode (Fig. 4).

**Fig. 5:**
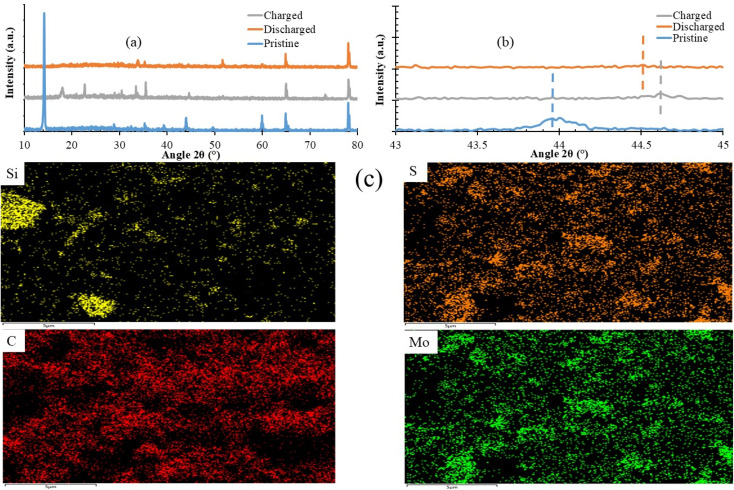
(a and b) XRD of the Si@Mo@S electrode and (c) elemental EDS mapping of the pristine form.

To follow the structure evolution at different electrochemical states, we studied the structural properties of the Mo@Si@S electrode through XRD, as shown in Fig. 5. For the pristine form for MoS_2_'s hexagonal phase structure, (002) and (006) planes refer to peaks at 14.5°, and 44.6°. The observed XRD peaks for Mo@Si@S in the charged form during the second cycle show the demolition of the peaks. This is related to the formation of polysulfide (K_2_S_*x*_) (K_2_S_3_, K_2_S_5_ and K_2_S_6_, K_2_S_2_, K_2_S_4_) compounds as charged compounds for charge storage and transfer.^37^ Those compounds remain stable in the discharge process, meaning molecules easily migrate through the Mo@Si@S cathode. For the peak at 44.6° for the (006) plane of MoS_2_, it is observed that there was a blue shift after the charging process, and it was slightly demolished after discharge to the pristine position. This is due to the broken inter-layer space between MoS_2_'s layers, and the K_2_S_*x*_ compounds are intercalated and diffused between those layers for storage. This indicates that the 2*θ*° position Mo@Si@S has significantly increased, and intensity decreases due to the migration of K ions from the electrolyte or the corroded anode. Hence, the K ratio increases and interacts with S in MoS_2_.^38,39^

The K metal anode is highly reactive with conventional carbonate electrolytes, which are almost the only electrolytes used in K ion batteries due to their high oxidative stability *vs.* K^+^/K. This limits the coulombic efficiency of potassium plating/stripping under 50%,^40^ which further contributes to capacity loss and low retention. Several tactics have been addressed to solve this issue. Among them, electrolyte modification and interface engineering are very promising in inhibiting uncontrollable dendrite formation and improving battery performance.^41^ For instance, Qin *et al.*^42^ employed an artificial stable SEI approach on the K anode while using Prussian blue PB Fe_4_[Fe(CN)_6_]_3_ and perylene-3,4,9,10-tetracarboxylic dianhydride (PTCDA) as cathode materials to be tested with 3 M KFSI and 1 M KPF_6_ in EC/DEC. They used pristine K as an anode where the pristine K‖PTCDA cell broke after only 31 cycles due to severe dendrite growth and the absence of a protective SEI, and as a result, electrolyte decomposition took place. They developed a continuous and compact potassium, the CCPP anode, as an alternative to pristine K. The CCPP‖PTCDA cell delivered 133.56 mA h g^−1^ and maintained a capacity of 117.5 mA h g^−1^ beyond 200 cycles with 88% capacity retention, indicating decreased electrolyte loss due to the artificial stable SEI. Similar performance enhancements were obtained using the Prussian blue cathode. These results indicate the significance of forming a protective SEI on K anode in the future optimization for practical application of the current Mo@Si@S electrode in potassium metal anode-based batteries.

## 4. Conclusion

In conclusion, Si@Mo@S electrodes with a fixed ratio of MoS_2_ and SiC mixed with S were prepared to be used as a K/S battery cathode with three different electrolytes. Although the KPF_6_ and KFSI cells show high initial capacity, the polysulfide shuttle within the Mo@Si@S electrode leads to the demolition of redox peaks after the 1^st^ cycle. Self-made electrolytes shows the stability of redox peaks until the 3^rd^ cycle before cycling stability was reduced owing to the growth of the interfacial barrier and polysulfide shuttle. KPF_6_ electrolytes showed the best capacity retention. The changes in *ex situ* XRD are related to the K anode corrosion and diffusion with S forming potassium polysulfide (K_2_S_*x*_), which makes diffusion within the cathode and causes sulfur shuttle, which is currently the major limitation. There are several optimizing strategies to overcome such challenges. The underlying mechanism of the polysulfide shuttle can be further investigated using simultaneous analysis of cell behavior by *operando* spectroscopy. Additionally, solid-state electrolytes are strongly expected to solve the issue of polysulfide shuttle and are recommended for integrating with liquid electrolytes and Mo@Si@S electrodes. The solid layer can form a protective layer for blocking the polysulfides while benefiting from the high ionic conductivity of liquid electrolytes. Finally, forming an artificial SEI on K metal can be highly beneficial in avoiding anode corrosion. This study has deepened our understanding and helped us investigate the electrolyte compatibility of a new cathode candidate to enhance the cycle life of K/S batteries, which can be further studied and optimized to build K-batteries and use this material as a cathode for commercial batteries.

## Data availability

All the data analyzed or generated during the study are available from the corresponding author upon request, Eslam Sheha.

## Conflicts of interest

There are no conflicts to declare.
